# Role of membrane glycerolipids in photosynthesis, thylakoid biogenesis and chloroplast development

**DOI:** 10.1007/s10265-016-0827-y

**Published:** 2016-04-25

**Authors:** Koichi Kobayashi

**Affiliations:** Department of Life Sciences, Graduate School of Arts and Sciences, The University of Tokyo, Komaba 3-8-1, Meguro-ku, Tokyo, 153-8902 Japan

**Keywords:** *Arabidopsis thaliana*, Chloroplast, Membrane lipid, Photosynthesis, Thylakoid membrane

## Abstract

The lipid bilayer of the thylakoid membrane in plant chloroplasts and cyanobacterial cells is predominantly composed of four unique lipid classes; monogalactosyldiacylglycerol (MGDG), digalactosyldiacylglycerol (DGDG), sulfoquinovosyldiacylglycerol (SQDG) and phosphatidylglycerol (PG). MGDG and DGDG are uncharged galactolipids that constitute the bulk of thylakoid membrane lipids and provide a lipid bilayer matrix for photosynthetic complexes as the main constituents. The glycolipid SQDG and phospholipid PG are anionic lipids with a negative charge on their head groups. SQDG and PG substitute for each other to maintain the amount of total anionic lipids in the thylakoid membrane, with PG having indispensable functions in photosynthesis. In addition to biochemical studies, extensive analyses of mutants deficient in thylakoid lipids have revealed important roles of these lipids in photosynthesis and thylakoid membrane biogenesis. Moreover, recent studies of *Arabidopsis thaliana* suggest that thylakoid lipid biosynthesis triggers the expression of photosynthesis-associated genes in both the nucleus and plastids and activates the formation of photosynthetic machineries and chloroplast development. Meanwhile, galactolipid biosynthesis is regulated in response to chloroplast functionality and lipid metabolism at transcriptional and post-translational levels. This review summarizes the roles of thylakoid lipids with their biosynthetic pathways in plants and discusses the coordinated regulation of thylakoid lipid biosynthesis with the development of photosynthetic machinery during chloroplast biogenesis.

## Introduction

Chloroplasts are prototypical members of plastids, the double-membrane organelles specific to plants and algae, with the ability to perform oxygenic photosynthesis. In addition to outer and inner envelope membranes, chloroplasts have an extensively developed internal membrane system, the thylakoid membrane, where photochemical and electron transport reactions take place. In the thylakoid membrane, thousands of proteins, pigments and photosynthetic cofactors are embedded in the bilayer formed by glycerolipids. Reflecting their evolutionary relationship, chloroplasts and cyanobacteria have similar lipid compositions in thylakoid membranes, with non-phosphorous glycolipids as major constituents (Table 1), although typical cellular membranes of animals, fungi, or non-photosynthetic bacteria use phospholipids for building blocks of lipid bilayers.

**Table 1:** Membrane lipid composition of plant plastids and cyanobacteria

		MGDG	DGDG	SQDG	PG	PI	PC	PE	Others
Chloroplast	Thylakoids^a^	53	27	7	7	2	0	0	4
	Inner envelope^b^	49	30	5	8	1	6	0	1
	Outer envelope^b^	17	29	6	10	5	32	0	1
Nongreen plastid^c^		31.5	27.5	6	9	4.5	20	1	0.5
*Synechocystis* sp. PCC 6803^d^		54	18	15	13	0	0	0	0

^a^Isolated from spinach leaves (wt%) (Dorne et al. 1990)

^b^Isolated from spinach leaves (wt%) (Block et al. 1983)

^c^Isolated from cauliflower buds (wt%) (Alban et al. 1988)

^d^Isolated from wild-type *Synechocystis* cells (mol%) (Wada and Murata 1989)

The lipid composition unique to chloroplasts and cyanobacteria implies a special requirement of these glycerolipids for oxygenic photosynthesis. Thylakoid lipids provide a lipid bilayer matrix for photosynthetic protein–cofactor complexes and support the electron transport chain in the thylakoid membrane. The lipid bilayer avoids the free diffusion of ions across the membrane and enables generation of a proton motive force via photosynthetic activities. Furthermore, glycerolipids function as structural components of several photosynthetic complexes in the thylakoid membrane and are directly and indirectly involved in photosynthetic reactions, as described later in brief and in detail in comprehensive reviews (Domonkos et al. 2008; Mizusawa and Wada 2012; Sato 2004).

Biosynthesis of the thylakoid membrane is a determinant process of chloroplast biogenesis and requires the coordinated synthesis of lipids with proteins, pigments and cofactors. Reflecting the fundamental function of lipids in thylakoid formation, the biosynthesis and homeostasis of lipids strongly affect chloroplast development and thereby the development of plants. By summarizing briefly the composition, biosynthetic pathways and photosynthetic functions of thylakoid lipids in plants and cyanobacteria, this review focuses on the regulatory aspects of thylakoid lipid biosynthesis in coordination with the development of photosynthetic machinery and chloroplast biogenesis in higher plants.

## Major lipid class in chloroplasts

**Fig. 1:**
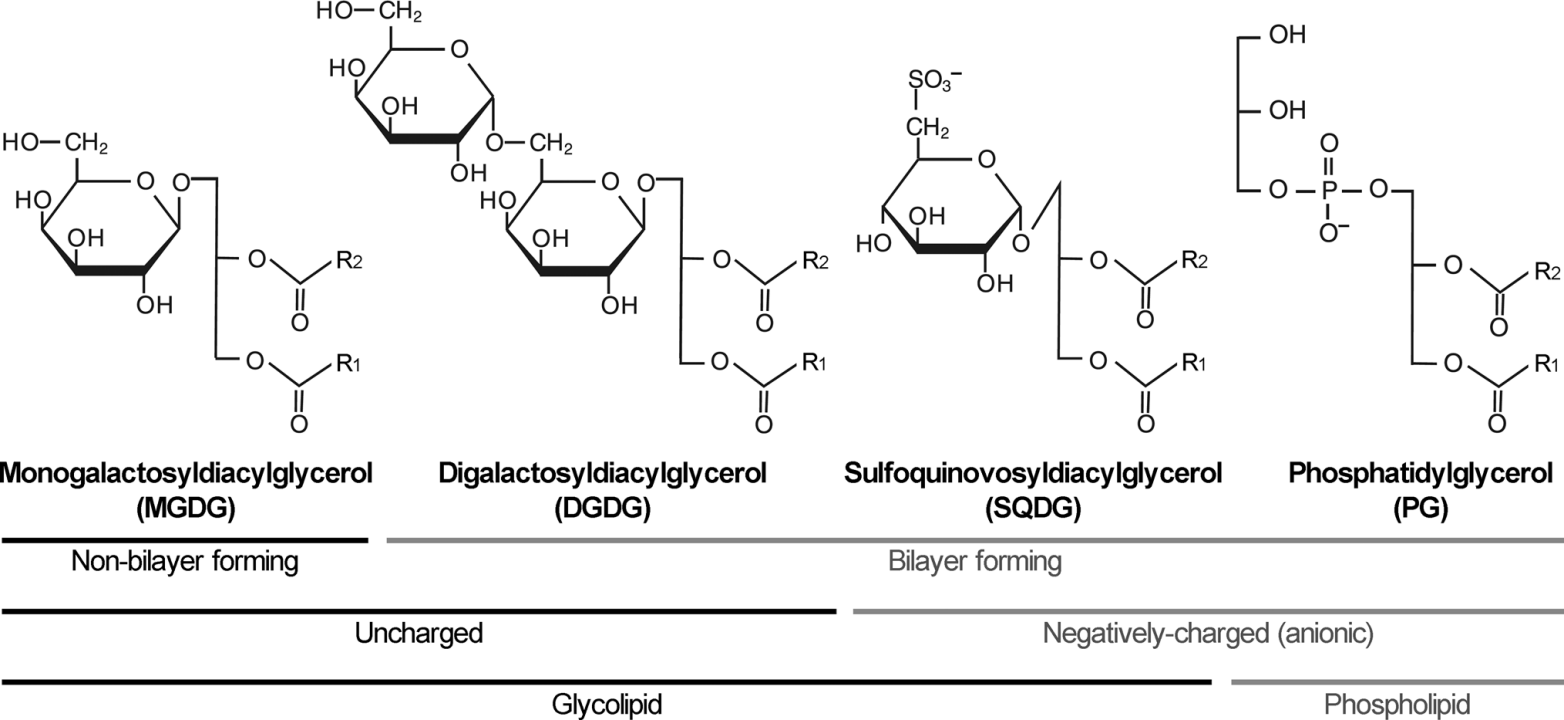
Structure and characteristic of glycerolipids in the thylakoid membrane. Major glycerolipids in the thylakoid membrane of plants and cyanobacteria can be classified into non-bilayer-forming (MGDG) and bilayer-forming lipids (DGDG, SQDG and PG), uncharged (MGDG and DGDG) and negatively-charged lipids (SQDG and PG), and glycolipids (MGDG, DGDG, SQDG) and phospholipids (PG).* R*_1_ and* R*_2_ denote hydrocarbon chains of fatty acids

As in animals, fungi, and many prokaryotes, phosphoglycerolipids constitute the major lipid fraction of biological membranes in plants (Moreau et al. 1998). However, as an exception, plastids have very unique lipid composition, with nonphosphorous mono- and digalactosyldiacylglycerols (MGDG and DGDG, respectively) constituting a major fraction (Table 1). Particularly, these galactolipids are predominant in the thylakoid membrane; MGDG and DGDG account for about 50 and 25 % of total thylakoid lipids, respectively (Dorne et al. 1990). The remainder consists of sulfoquinovosyldiacylglycerol (SQDG) and phosphatidylglycerol (PG), which are anionic lipids with a negative charge in their head groups (Fig. 1). Although PG is the sole phospholipid in cyanobacteria (Wada and Murata 1998), another phospholipid, phosphatidylinositol is found as a minor constituent of the thylakoid membrane in plants (Table 1) (Dorne et al. 1990). Some cyanobacteria species feature monoglucosyldiacylglycerol (GlcDG) as a major lipid class (Sato 2015), which is absent in plants because of a difference in galactolipid biosynthetic pathways between plants and cyanobacteria as described later.

In plant chloroplasts, the lipid composition of the inner envelope membrane is similar to that of the thylakoid membrane but different from that of the outer envelope membrane, where phosphatidylcholine represents a large proportion (Block et al. 1983) (Table 1). Galactolipids and SQDG are also the main membrane constituents in non-green plastids (Alban et al. 1988), although these lipids are scarcely detected in extraplastidic membranes of plant cells under standard growth conditions (Moreau et al. 1998). As an exception, DGDG greatly accumulates in extraplastidic membranes such as plasma membranes (Andersson et al. 2003), mitochondrial membranes (Jouhet et al. 2004) and tonoplasts (Andersson et al. 2005) under phosphate (P)-deficient conditions, presumably to economize the use of phosphorus in membranes.

Membrane glycerolipids can be classified as non-bilayer-forming and bilayer-forming lipids by their physical properties in aqueous dispersions (Fig. 1) (Jouhet 2013). MGDG has a cone-like shape with a small head group and broadened poly-unsaturated fatty acid tails. This geometry allows MGDG to form the hexagonal-II phase with its polar head group facing toward the center of inverted micellar tubules (Shipley et al. 1973). Small curvature lipids such as DGDG, SQDG and PG have more cylindrical shapes and form lamellar bilayer phases in mixtures with water (Jouhet 2013). The ratio of non-bilayer forming MGDG to bilayer-forming lipids such as DGDG affects the global and local phase behavior of lipid bilayers (Demé et al. 2014) and is tightly regulated in response to various stresses to maintain membrane structures and enzymatic activities (Moellering and Benning 2011).

The amount of total anionic lipids in the thylakoid membrane is strictly maintained in plants and cyanobacteria. Both in cyanobacteria and plants, the SQDG content increases in response to P deficiency to compensate for reduced PG content (Essigmann et al. 1998; Güler et al. 1996). Conversely, loss of SQDG in *Synechococcus* sp. PCC 7942 (Güler et al. 1996), *Synechocystis* sp. PCC 6803 (hereafter *Synechocystis*) (Aoki et al. 2004), *Chlamydomonas reinhardtii* (Sato et al. 1995b), and *Arabidopsis* (Yu et al. 2002) by genetic disruption increased PG content, to maintain a constant amount of total anionic lipids. A similar phenomenon was observed in a purple photosynthetic bacterium, *Rhodobacter sphaeroides*, with P deficiency (Benning et al. 1993), which suggests a universal requirement for keeping the amount of anionic lipids constant in membranes of photosynthetic organisms.

## Biosynthesis of thylakoid membrane lipids in plants

**Fig. 2:**
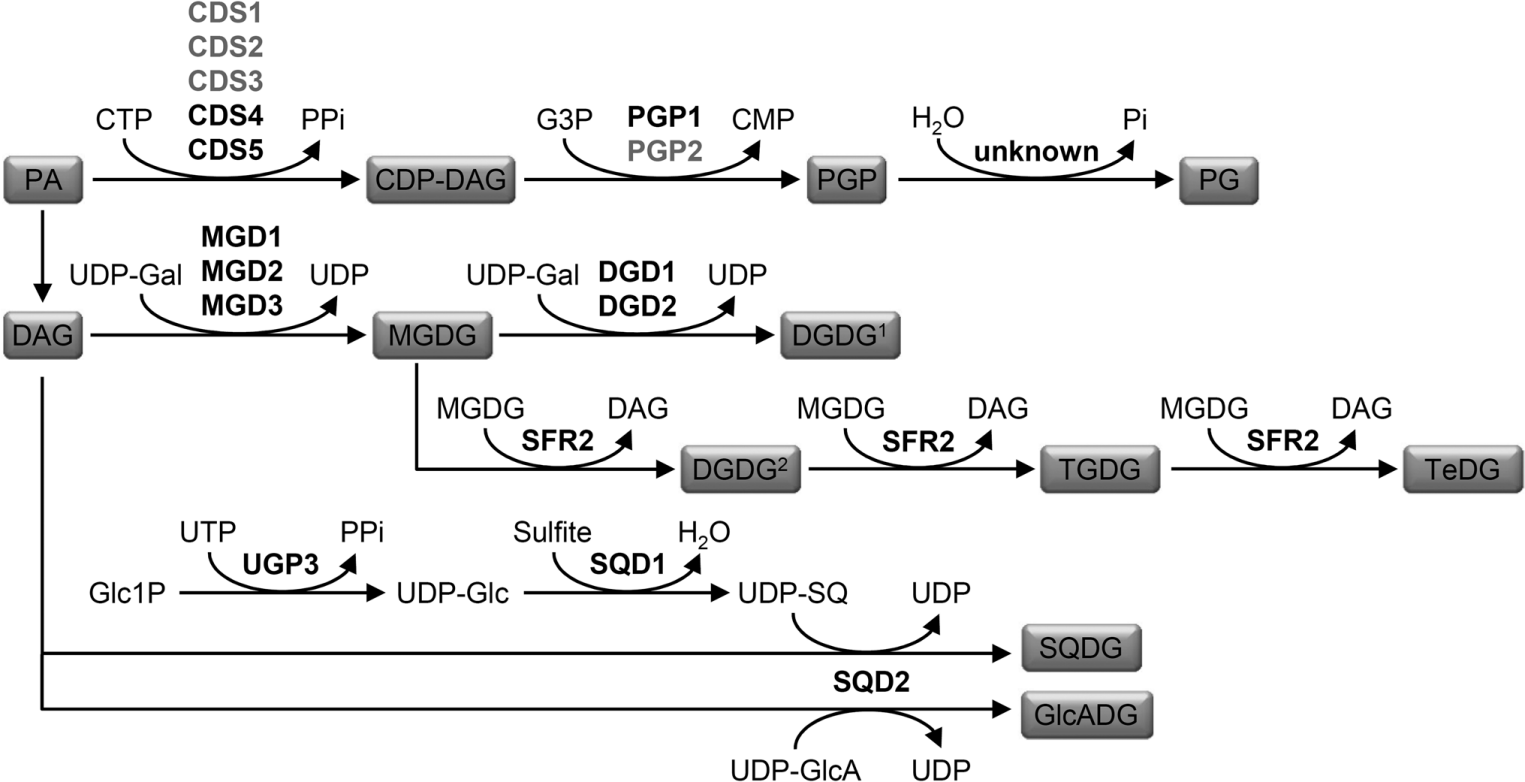
Biosynthetic pathway of thylakoid glycerolipids from phosphatidic acid (PA) in *Arabidopsis thaliana*. Lipid products and intermediates are shown in a* box*. Enzymes for each step are in* bold*; enzymes in *black* are localized in the plastid, whereas those in *gray* are in the endoplasmic reticulum. *CDP* cytidine 5′-diphosphate; *CMP* cytidine 5′-monophosphate; *CTP* cytidine 5′-triphosphate; *DAG* diacylglycerol; *DGDG* ^*1*^ α,β-digalactosyldiacylglycerol; *DGDG* ^*2*^ β, β-digalactosyldiacylglycerol; *Gal* galactose; *Glc* glucose; *GlcA* glucuronic acid; *GlcADG* glucuronosyldiacylglycerol; *Glc1P* glucose 1-phosphate; *G3P* glycerol 3-phosphate; *MGDG* monogalactosyldiacylglycerol; *PG* phosphatidylglycerol; *PGP* phosphatidylglycerophosphate; *PPi* pyrophosphate; *SQ* sulfoquinovose; *SQDG* sulfoquinovosyldiacylglycerol; *TeDG* tetragalactosyldiacylglycerol; *TGDG* trigalactosyldiacylglycerol; *UDP* uridine 5′-diphosphate

Most of the genes involved in thylakoid lipid biosynthesis in higher plants have been identified and characterized by genetic and reverse genetic analyses in *Arabidopsis* (Fig. 2). Plants synthesize MGDG by using UDP-galactose in one step (Joyard et al. 1998), whereas cyanobacteria first synthesize GlcDG by using UDP-glucose and then epimerize it to MGDG (Awai et al. 2006, 2014; Sato and Murata 1982). A study of rice showed that a chloroplast-localized UDP-glucose epimerase, PHOTOASSIMILATE DEFECTIVE 1, converts UDP-glucose to UDP-galactose in the stroma, some of which is used for MGDG biosynthesis (Li et al. 2011) . MGDG synthase in plastid envelopes transfers a galactose from UDP-galactose to diacylglycerol in the β-configuration to form MGDG (Shimojima et al. 1997). Three MGDG synthase paralogs (MGD1, MGD2, and MGD3) were identified in *Arabidopsis* (Awai et al. 2001; Miège et al. 1999); inner envelope-localized MGD1 is the major isoform responsible for most MGDG synthesis in chloroplasts (Jarvis et al. 2000; Kobayashi et al. 2007), whereas outer envelope-localized MGD2 and MGD3 specifically function under P-limited conditions mainly to supply MGDG to DGDG biosynthesis as a substrate (Kobayashi et al. 2004, 2009a). DGDG synthase in the plastid outer-envelope membrane transfers a second galactose to MGDG from UDP-galactose in the α-configuration to form (αβ)DGDG (Froehlich et al. 2001; Kelly and Dörmann 2002; Kelly et al. 2003). In *Arabidopsis* and other angiosperms, two isoforms, DGD1 and DGD2, have been identified for DGDG synthase (Dörmann et al. 1999; Gaude et al. 2004; Kelly and Dörmann 2002); DGD1 is responsible for most of the DGDG synthesis in chloroplasts in combination with MGD1, whereas DGD2 contributes to the alternative pathway with MGD2 and MGD3 (Benning and Ohta 2005; Kobayashi et al. 2009b). In addition, another enzyme, SENSITIVE TO FREEZING 2 (SFR2), functions to form DGDG and oligogalactolipids in the plastid envelope membrane. SFR2 transfers the galactose head group from MGDG to another galactolipid in the β-configuration to form (ββ)DGDG and successively tri- and tetragalactosyldiacylglycerol (Fig. 2) (Moellering et al. 2010). This enzyme is required for freezing tolerance in cold-acclimated conditions presumably via stabilizing the chloroplast membrane by increasing the hydration interacted with the membrane and by remodeling the ratio of bilayer-forming to non-bilayer-forming lipids (Moellering et al. 2010).

SQDG biosynthesis takes place in the plastids, starting from the formation of UDP-glucose from glucose 1-P and UTP by a plastidic UDP-glucose pyrophosphorylase (UGP3) (Okazaki et al. 2009). Then, UDP-glucose is converted to UDP-sulfoquinovose (SQ) with sulfite by UDP-SQ synthase (SQD1). In the last step, the SQ moiety of UDP-SQ is transferred to diacylglycerol by SQDG synthase (SQD2) to form SQDG. Both SQD1 and SQD2 are encoded by single loci in the *Arabidopsis* genome (Essigmann et al. 1998; Okazaki et al. 2009; Yu et al. 2002). Knockout mutations in *UGP3*, *SQD1*, and *SQD2* all resulted in complete loss of SQDG in *Arabidopsis*, which demonstrates indispensable roles of these genes in SQDG biosynthesis. SQD2 is also involved in the formation of another acidic glycolipid, glucuronosyldiacylglycerol (GlcADG), under P-deficient conditions presumably by catalyzing transfer of glucuronic acid to diacylglycerol. GlcADG accumulates in response to P deficiency and may play a role in adaptation to P limitation, although its function and subcellular localization remain unclear (Okazaki et al. 2013).

In plant cells, PG is synthesized in plastids, mitochondria and endoplasmic reticulum (ER) membranes (Wada and Murata 2007). In the first step, CDP-diacylglycerol is formed from phosphatidic acid (PA) and CTP by CDP-diacylglycerol synthase (CDS). Five genes, *CDS1* to *CDS5*, have been identified as functional CDSs in *Arabidopsis*; CDS1, CDS2 and CDS3 are targeted to ER membranes (Zhou et al. 2013), and CDS4 and CDS5 are localized in plastids (Haselier et al. 2010). Then CDP-diacylglycerol is converted to PG phosphate (PGP) with glycerol 3-P by PGP synthase. Two isoforms (PGP1 and PGP2) for PGP synthase have been identified in *Arabidopsis*; PGP1 is dual-localized in plastids and mitochondria (Babiychuk et al. 2003), whereas PGP2 is targeted to the ER (Tanoue et al. 2014). Knockout mutations in PGP1 strongly impaired thylakoid membrane development with greatly reduced PG content (Babiychuk et al. 2003; Hagio et al. 2002; Kobayashi et al. 2015), whereas the *pgp2* single mutant showed no obvious growth defects. However, a *pgp1pgp2* double mutant was embryonic-lethal with no PG synthesizing activity (Tanoue et al. 2014). Thus, PGP1 is responsible for PG biosynthesis in the chloroplast and PGP2 has a subsidiary role. However, loss of PGP1 did not affect mitochondria features (Babiychuk et al. 2003; Hagio et al. 2002), so ER-localized PGP2 can complement the PGP1 function in mitochondria. The last step for PG biosynthesis is dephosphorylation of PGP by PGP phosphatase. A gene encoding a putative PGPP, with sequence similarity to a PGPP (*pgpB*) of *Escherichia coli*, was reported to be involved in PG biosynthesis in *Anabaena* sp. PCC 7120 (Wu et al. 2006). However, homologous genes of *Anabaena pgpB* in *Synechocystis* and *Arabidopsis* encode PA phosphatase (Nakamura et al. 2007). Meanwhile, Hung et al. (2015) reported that a *Chlamydomonas* lipid phosphatase gene (*CrPGPP1*) homologous to the yeast PGPP (Gep4p) could complement a yeast PGPP mutant (*Δgep4*), which suggests that CrPGPP1 is a functional PGPP in *Chlamydomonas*. *Arabidopsis* has a gene (At3g58830) that is homologous to CrPGPP, and future studies are anticipated to reveal genes responsible for this step in higher plants.

## Role of glycerolipids in thylakoid biogenesis

**Fig. 3:**
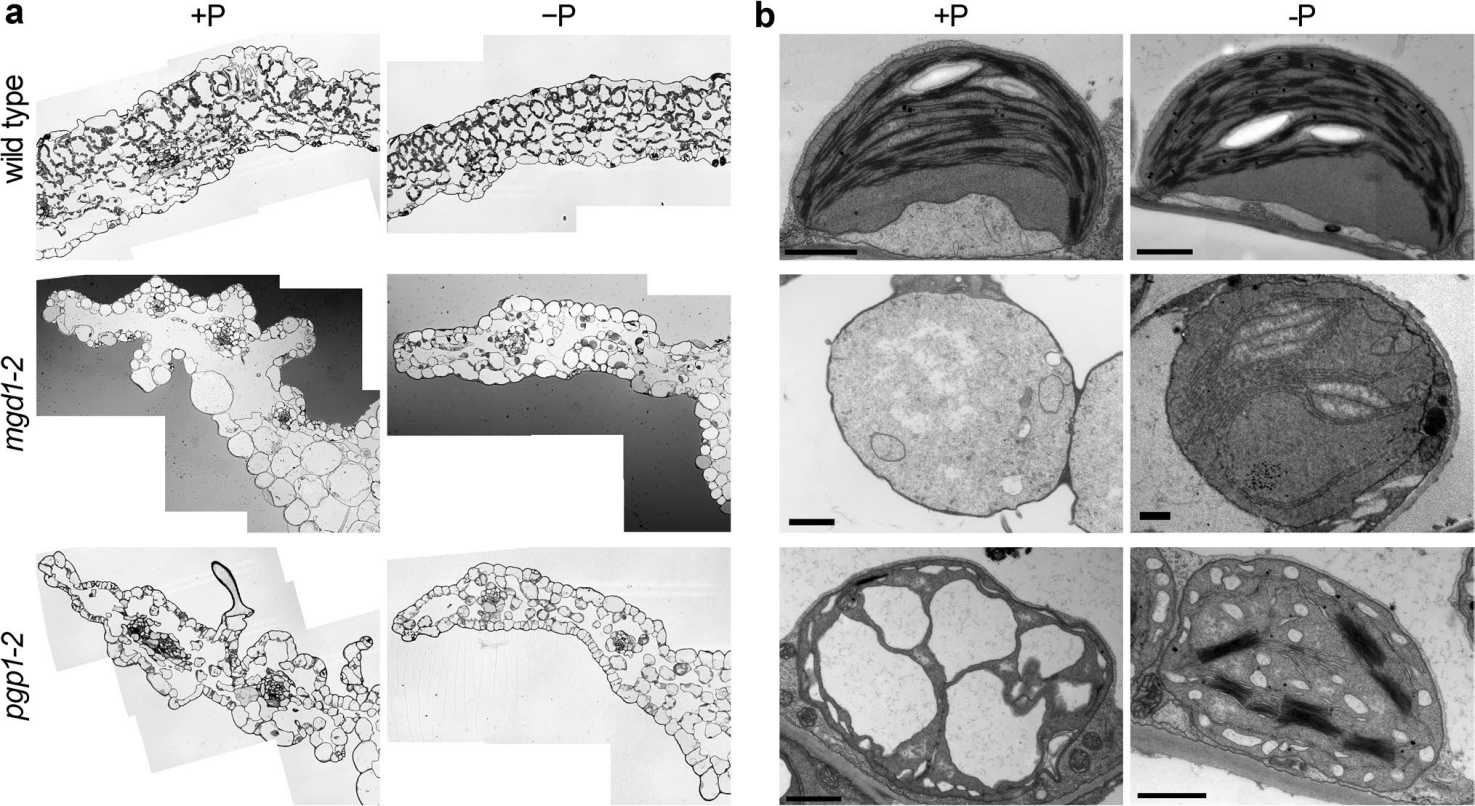
**a** Leaf morphology and **b** ultrastructure of leaf plastids in thylakoid lipid mutants of *Arabidopsis thaliana* under phosphate-sufficient (+P) or -deficient (−P) conditions. Images of wild type and *pgp1*-*2* in **a** and **b** are adapted from Kobayashi et al. (2015) and those of *mgd1*-*2* plastids in **b** are from Kobayashi et al. (2013) with permission of Springer and John Wiley and Sons, respectively, via Copyright Clearance Center. Leaf section images of *mgd1*-*2* in **a** were kindly provided by Kiminori Toyooka, Mayuko Sato and Mayumi Wakazaki, RIKEN CSRS, Japan. *Bars* in **b** = 1 μm

Glycerolipids provide a lipid bilayer matrix for photosynthetic protein–cofactor complexes, so lipid composition greatly affects the structure and characteristics of the thylakoid membrane. Partial deficiency of MGDG decreased the amount of thylakoid membrane, with altered architecture, in *Arabidopsis* (Fujii et al. 2014; Jarvis et al. 2000), tobacco (Wu et al. 2013) and maize (Myers et al. 2011). A knockout mutation (*mgd1*-*2*) of *Arabidopsis* *MGD1* by a T-DNA insertion resulted in severe loss of the thylakoid membrane along with a substantial reduction in content of both galactolipids (Kobayashi et al. 2007). Instead of thylakoid membrane networks, invaginated inner-envelope membranes were observed together with immature vesicular structures in *mgd1*-*2* plastids (Kobayashi et al. 2007, 2013). Specific inhibition of MGDG synthesis by the inhibitor galvestine-1 also caused invaginated structures from the inner envelope membrane (Botté et al. 2011). Considering that the invagination of the inner envelope membrane is observed only at the initial stage of chloroplast differentiation in wild-type plants (Vothknecht and Westhoff 2001), the invaginated membranes with impaired MGDG synthesis may reflect intermediate structures of an early process of thylakoid biogenesis. Under P-deficient conditions, *MGD2* and *MGD3* were strongly upregulated in the *mgd1*-*2* mutant and produced DGDG in collaboration with DGDG synthase, which resulted in the formation of immature thylakoid membranes in the mutant plastids (Fig. 3) (Kobayashi et al. 2013). Moreover, inner envelope invagination actively occurred in the plastids of P-deficient *mgd1*-*2* leaves and was occasionally associated with laminated internal membranes, which supports that envelope invagination is somehow linked with thylakoid membrane biogenesis. Meanwhile, identification and characterization of putative chloroplast-localized vesicle transport proteins in *Arabidopsis* suggest that vesicle transport systems play a role in thylakoid biogenesis (Karim and Aronsson 2014; Rast et al. 2015). Further studies are required to elucidate the relationship between membrane glycerolipids and vesicle transport in the process of thylakoid biogenesis.

DGDG is important for the proper formation and maintenance of the thylakoid membrane; in DGDG-deficient *Arabidopsis* mutants, the thylakoid membrane of leaf chloroplasts was strongly bent, with large thylakoid-free areas in the stroma (Dörmann et al. 1995; Hölzl et al. 2009). The distorted thylakoid structure in the mutants was recovered by transgenic expression of a bacterial glucosyltransferase and consequent accumulation of glucosylgalactosyldiacylglycerol (Hölzl et al. 2009). As proposed by an in vitro analysis showing that DGDG affects the membrane adhesion via hydrogen bonds between polar heads of adjacent bilayers (Demé et al. 2014), disaccharide head groups of DGDG would play a crucial role in the thylakoid architecture. In addition, the ratio of bilayer-forming DGDG to non-bilayer-forming MGDG could affect the properties and structures of thylakoid membranes by altering the lipid bilayer from hexagonal II to lamellar phases (Demé et al. 2014).

Development of the thylakoid membrane is also strongly affected by the acidic lipids PG and SQDG. Deficiency of chloroplast PG in *Arabidopsis* mutants resulted in the formation of vesicles, enlarged vacuolated structures, or underdeveloped membrane fragments (Fig. 3) (Babiychuk et al. 2003; Hagio et al. 2002; Haselier et al. 2010; Kobayashi et al. 2015). The role of PG in thylakoid membrane biogenesis was revealed by using a T-DNA insertional PGP1 knockout mutant (*pgp1*-*2*), in which the level of total cellular PG was decreased by ~80 % as compared with the wild type. Under P-deficient conditions, the proportion of galactolipids and SQDG was substantially increased in *pgp1*-*2*, which led to the development of the thylakoid membrane in the mutant. However, *pgp1*-*2* chloroplasts showed many vesicular membrane structures under P-deficiency, which are barely detected in wild-type chloroplasts (Fig. 3), which suggests a special requirement of PG for internal membrane organization in chloroplasts. In contrast to the strong effect of PG deficiency on thylakoid biogenesis, lack of SQDG biosynthesis in *Arabidopsis* had no effect on the thylakoid architecture. Only together with a small reduction in PG content by the leaky *pgp1*-*1* mutation, loss of SQDG decreased grana stacking in leaf chloroplasts (Yu and Benning 2003). Meanwhile, in *Chlamydomonas*, deficiency of SQDG caused strong curling of thylakoid membranes with little effect on grana stacking (Sato et al. 1995b). Hennig et al. (2015) reported that the *Synechocystis* IM30 protein, a homolog of VESICLE-INDUCING PROTEIN IN PLASTIDS 1 involved in chloroplast membrane maintenance in plants (Zhang and Sakamoto 2015), specifically binds to membranes containing PG and SQDG and induces membrane fusion. Thus, chloroplast anionic lipids may participate in membrane organization through such protein complexes.

## Role of thylakoid membrane lipids in photosynthesis

Thylakoid lipids, not only providing a lipid bilayer matrix, are also required for photosynthetic activities in plants, algae and cyanobacteria at multiple levels. X-ray crystallography studies demonstrate that thylakoid lipids are involved as structural components in photosystem II (PSII) (Guskov et al. 2009; Umena et al. 2011) and PSI complexes (Jordan et al. 2001; Qin et al. 2015). Moreover, mutant analyses in various photosynthetic organisms have been elucidated the specific roles of lipids in oxygenic photosynthesis (Mizusawa and Wada 2012).

In a knockdown mutant of *Arabidopsis* *MGD1* (*mgd1*-*1*), a ~40 % decrease in MGDG level had no effect on intrinsic PSII activity but increased conductivity of the thylakoid membrane, which impaired thylakoid membrane energization and photoprotection (Aronsson et al. 2008). A similar loss of MGDG in a tobacco *MGD1* mutant reduced levels of the cytochrome *b*_6_ *f* complex and blocked intersystem electron transport (Wu et al. 2013). In *Arabidopsis* transgenic plants expressing an artificial microRNA targeting *MGD1* (*amiR*-*MGD1*), a further decrease in MGDG level (~80 % from wild-type levels) with partial DGDG reduction strongly impaired the PSII photochemical reaction and energy coupling between reaction centers and antenna complexes (Fujii et al. 2014). Moreover, critical loss of MGDG (~95 % from wild-type levels) caused complete loss of PSII activity with disrupted formation of photosystem complexes (Kobayashi et al. 2013). In addition to these results in vivo, in vitro analyses revealed that MGDG is required for ordered oligomerization of light-harvesting complex II (LHCII) (Schaller et al. 2011) and dimerization of PSII (Kansy et al. 2014) and enhances energy coupling between LHCII and PSII core complexes (Zhou et al. 2009). MGDG also plays a role in photoprotective nonphotochemical quenching. With its ability to form a hexagonal lipid phase, MGDG mediates solubilization of violaxanthin and the binding of violaxanthin de-epoxidase to the thylakoid membrane (Jahns et al. 2009). Specifically, an MGDG phase surrounding the LHCII strongly enhances the de-epoxidation of LHCII-associated violaxanthin in the thylakoid membrane (Schaller et al. 2010).

DGDG plays specific roles in oxygenic photosynthesis. Disruption of DGDG biosynthesis in the *Synechocystis* *dgdA* mutant caused dissociation of extrinsic proteins of PSII, thereby destabilizing the oxygen-evolving complex (Sakurai et al. 2007b). Moreover, loss of DGDG in *Synechocystis* increased the sensitivity to photoinhibition under high temperature and high light, particularly by impairing the repair cycle of PSII (Mizusawa et al. 2009a, b). Analyses of *Arabidopsis* mutants deficient in DGDG biosynthesis revealed that DGDG is important for the structure and function of PSII (Härtel et al. 1997; Hölzl et al. 2006, 2009), primarily on the donor side (Reifarth et al. 1997; Steffen et al. 2005), and for trimerization of LHCII (Hölzl et al. 2006). DGDG deficiency enhanced photoinhibition under high light in *Arabidopsis* (Hölzl et al. 2009), so this lipid is required for photoprotection as in *Synechocystis*. DGDG also plays a crucial role in the function and stability of the PSI complex (Guo et al. 2005; Ivanov et al. 2006). Because biosynthesis of glucosylgalactosyldiacylglycerol instead of DGDG in chloroplasts could not fully restore the PSII functionality and trimerization of the LHCII complex (Hölzl et al. 2006, 2009), the second galactose of DGDG would have a specific role in the function of photosystem complexes.

Despite the requirement of galactolipids in oxygenic photosynthesis as described previously, the identification and characterization of a *Synechocystis* mutant lacking the epimerase converting GlcDG to MGDG revealed that the accumulation of GlcDG instead of MGDG and DGDG was not completely but was largely sufficient for photosynthetic activities and development of the thylakoid membrane structure (Awai et al. 2014). The data indicate that galactolipids are not a prerequisite for oxygenic photosynthesis. In fact, some cyanobacteria species accumulate a large amount of GlcDG under standard growth conditions (Sato 2015). However, unlike cyanobacteria, plants do not synthesize GlcDG, and whether GlcDG can substitute galactolipids in plant chloroplasts remains elusive.

A critical involvement of PG in photosynthesis was first reported in phospholipase-treated thylakoid membranes with specifically degraded PG. Degradation of PG by phospholipases impaired electron transport in PSII without decreasing PSI activity in pea and spinach thylakoids (Droppa et al. 1995; Jordan et al. 1983). Moreover, PG degradation caused dissociation of PSII dimer (Kruse et al. 2000), LHCII trimers (Nussberger et al. 1993), and the PSII–LHCII complexes (Kim et al. 2007) into monomeric forms. Comprehensive analyses of PG-deficient mutants in *Synechocystis*, *Chlamydomonas* and *Arabidopsis* have further provided deep insights into the role of PG in photosynthesis. Characterization of PG-deficient mutants in *Synechocystis* revealed that PG is indispensable for various processes of photosynthesis such as electron transport from the primary plastoquinone (Q_A_) to the secondary plastoquinone (Q_B_) at the PSII acceptor side (Endo et al. 2015; Gombos et al. 2002; Hagio et al. 2000; Itoh et al. 2012), reactivation of PSII after photoinhibition (Sakurai et al. 2003), maintenance of the oxygen-evolving complex activity at the PSII donor side (Sakurai et al. 2007a), and trimerization and activity of the PSI complex (Domonkos et al. 2004). Analysis of mutants in *Chlamydomonas* showed that PG is required for the synthesis of PSII core proteins (D1 and CP47) (Pineau et al. 2004), trimer formation of LHCII, state transition ability, and oxygen evolution activity (Dubertret et al. 1994; El Maanni et al. 1998). In the *Arabidopsis pgp1*-*2* mutant, the photochemical efficiency of PSII was greatly decreased, and further depletion of PG by phosphate starvation caused complete loss of the PSII activity (Kobayashi et al. 2015). In-depth analysis of the *pgp1*-*2* mutant further revealed the necessity for PG for electron transfer within PSII and cyclic electron transport around PSI (Kobayashi et al. 2016). Meanwhile, a point mutation in the *PGP1* gene (*pgp1*-*1*), which resulted in 30 % reduction in the total PG content with 80 % reduction in plastidic PGP activity, caused no remarkable defects in photochemical and electron transport activities (Xu et al. 2002; Yu and Benning 2003). In the *pgp1*-*1* mutant, the quality and quantity of photosynthetic components may be fine-tuned to the reduced PG content to maintain photosynthetic efficiency with support from another anionic lipid, SQDG. However, in the *pgp1*-*2* mutant, the stress with critical loss of PG in chloroplasts might exceed the homeostatic capacity of plants and cause fatal damage to photosynthetic machineries.

In contrast to the universal role of PG in photosynthesis, the requirement for SQDG greatly differs among organisms. In *Chlamydomonas* (Minoda et al. 2002, 2003; Sato et al. 1995a) and *Synechocystis* (Aoki et al. 2004), SQDG is required for activity of PSII at both donor and acceptor sides, with no crucial roles in PSI activity, but is not important for photosynthesis in *Synechococcus* sp. PCC 7942 (Aoki et al. 2004; Güler et al. 1996). In *Arabidopsis*, SQDG is dispensable under nutrient-sufficient conditions (Yu et al. 2002) but plays a role in maintaining the amount of total anionic lipids in photosynthetic membranes when PG content is decreased with P deficiency (Essigmann et al. 1998; Yu and Benning 2003). In fact, loss of SQDG by the *sqd2* knockout mutation strongly reduced actual PSII quantum efficiency only when PG content was decreased with the *pgp1*-*1* mutation at the same time (Yu and Benning 2003).

## Role of thylakoid membrane lipids in regulating chloroplast biogenesis

**Fig. 4:**
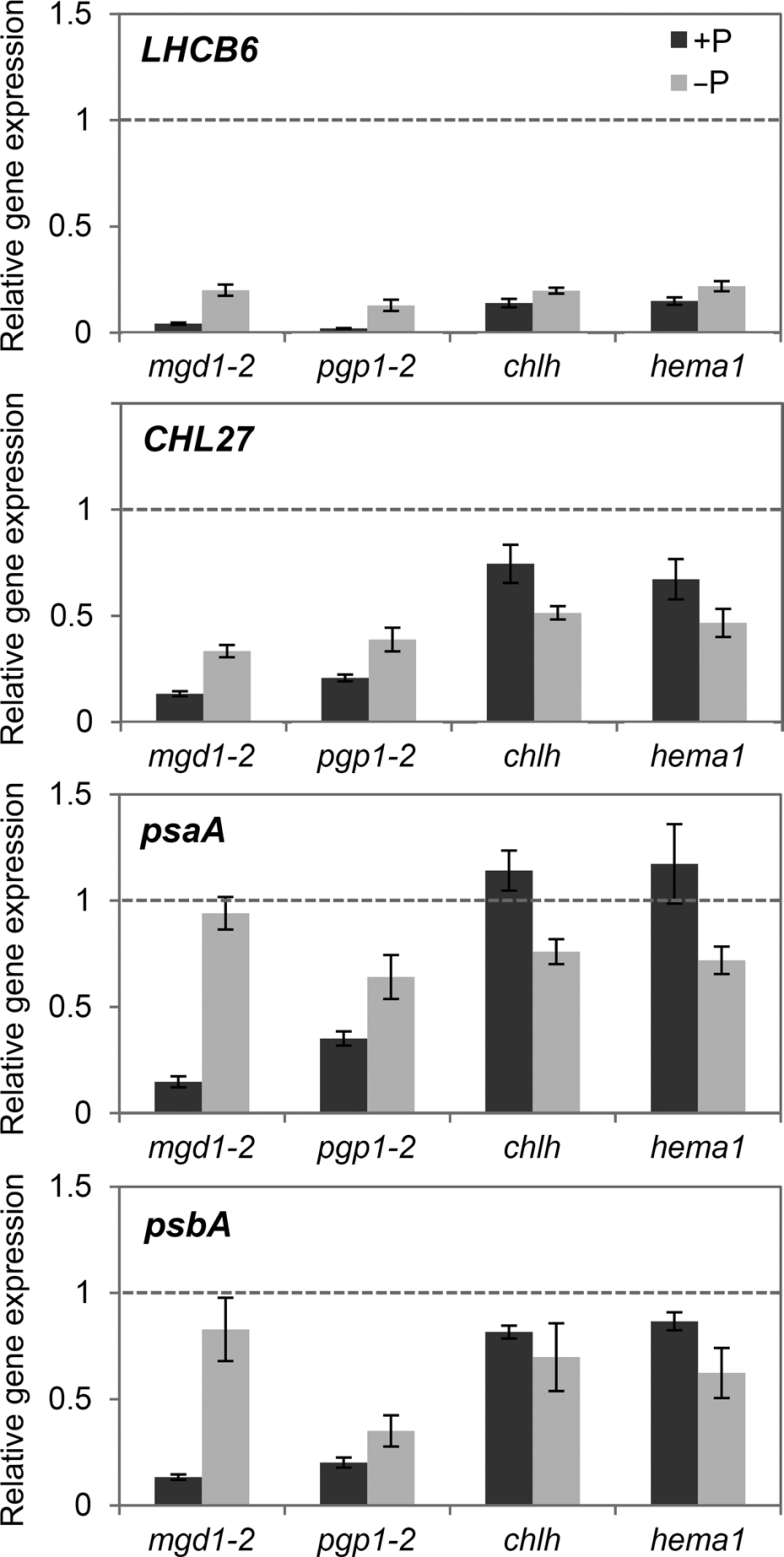
Expression of photosynthesis-associated genes in mutants for thylakoid lipid biosynthesis (*mgd1*-*2* and *pgp1*-*2*) and chlorophyll biosynthesis (*chlh* and *hema1*) under phosphate-sufficient (+P) or-deficient (−P) conditions. *LHCB6* and *CHL27* are nuclear genes encoding light-harvesting chlorophyll binding protein 6 and the membrane subunit of Mg-protoporphyrin IX monomethyl ester cyclase, respectively. *psaA* and *psbA* are plastid genes encoding core proteins of PSI and PSII, respectively. Data are presented as fold difference from the wild-type grown under +Pi conditions (*dotted lines*). Data in *mgd1*-*2*, *chlh* and *hema1* are adapted from Kobayashi et al. (2013) and those in *pgp1*-*2* are from Kobayashi et al. (2015) with permission of John Wiley and Sons and Springer, respectively, via Copyright Clearance Center

Photosynthesis with highly photoreactive chlorophylls involves a potential risk of photooxidative damage to the system. To prevent photodamage from immature or unbalanced electron transfer complexes, plants strictly regulate the formation of photosynthetic systems by orchestrating myriad components with the biogenesis of the thylakoid lipid bilayer. Transcriptional regulation of photosynthesis-related genes in response to thylakoid lipid biosynthesis would be one of the coordination mechanisms. In *Arabidopsis*, severe loss of galactolipids by the *mgd1*-*2* mutation strongly decreased the expression of photosynthesis-associated genes in the nucleus and plastids (Fig. 4) (Kobayashi et al. 2013). Similar results were observed on knocking down *MGD1* expression by *amiR*-*MGD1* during an early stage of chloroplast development (Fujii et al. 2014). However, downregulation of photosynthesis-associated genes in the *mgd1*-*2* mutant was weakened by activation of MGD2/3-mediated galactolipid biosynthesis with P deficiency and subsequent development of thylakoid-like membrane structures in leaf plastids (Fig. 4) (Kobayashi et al. 2013). A similar phenomenon was observed in the *pgp1*-*2* mutant; expression of photosynthesis-associated genes in the nucleus and plastids was downregulated under nutrient-sufficient conditions but was recovered along with biogenesis of internal membranes in plastids by P deficiency (Fig. 4) (Kobayashi et al. 2015). In the case of *pgp1*-*2*, activation of SQDG biosynthesis in response to P deficiency in addition to galactolipid biosynthesis would be important to compensate PG deficiency for thylakoid membrane biogenesis. By contrast, mutants of chlorophyll biosynthesis genes did not show upregulated photosynthesis-associated genes with P deficiency (Fig. 4) (Kobayashi et al. 2013), so activation of glycolipid biosynthesis and subsequent thylakoid development is indeed important for photosynthetic gene expression in thylakoid lipid mutants. Because photosynthetic electron transport was dysfunctional both in the *mgd1*-*2* and *pgp1*-*2* mutant even under P-deficient conditions (Kobayashi et al. 2013, 2015), upregulation of photosynthesis-associated genes in these mutants is independent of photosynthetic activities.

In mature chloroplasts, many tiny nucleoids are attached to the thylakoid membrane presumably via nucleoid-associated thylakoid proteins, which suggests an involvement of thylakoid formation for nucleoid distribution and morphology (Powikrowska et al. 2014). In fact, the inhibition of thylakoid biogenesis in the *mgd1*-*2* mutant caused a large aggregation of nucleoids in leaf plastids (Kobayashi et al. 2013). Similar to *mgd1*-*2*, *pgp1*-*2* showed a slight increase in nucleoid size (Kobayashi et al. 2015). Thus, lipid biosynthesis and subsequent thylakoid biogenesis may strongly affect nucleoid morphologic features in chloroplasts. Sekine et al. (2002) reported that the structural organization of plastid DNA is involved in regulating plastid transcription. Therefore, changes in nucleoid morphologic features in response to lipid biosynthesis may be linked to transcriptional activities of plastid-encoded genes, as was observed in down- and upregulation of plastid-encoded photosynthetic genes in response to inhibition and induction of thylakoid membrane formation, respectively, in *mgd1*-*2* (Kobayashi et al. 2013) and *pgp1*-*2* (Kobayashi et al. 2015). Because expression of plastid-encoded photosynthetic genes strongly affects the expression of nuclear-encoded photosynthesis-associated genes via retrograde signaling (Woodson and Chory 2008), thylakoid lipid biosynthesis may change the expression of photosynthesis-associated nuclear genes via the plastid-gene expression-associated signaling pathway. However, various processes in chloroplasts such as tetrapyrrole and carotenoid biosynthesis and redox changes also function as sources of plastid signals (Woodson and Chory 2008) and further studies are needed to reveal signaling pathways regulating photosynthesis-associated nuclear genes in response to thylakoid lipid biosynthesis.

## Role of thylakoid membrane lipids in cell and leaf development

During cell development, a tight metabolic coordination occurs between chloroplasts and other organelles. At the onset of seed germination, peroxisomes provide carbon and energy sources for heterotrophic growth by converting the fatty acids of triacylglycerols to succinate via β-oxidation and the glyoxylate cycle, together with mitochondrial metabolism and cytosolic gluconeogenesis (Graham 2008). However, in parallel with the activation of photosynthesis in cotyledon cells under light, mitochondria and peroxisomes change their function to operate photorespiratory metabolism, which recycles 2-phosphoglycolate, the product of oxygenation reaction instead of carboxylation by Rubisco in chloroplasts (Peterhansel et al. 2010). Thus, plant cells need to coordinate functional differentiation of these organelles with chloroplast development during the transition from heterotrophic to photoautotrophic growth.

The involvement of galactolipid biosynthesis in coordinated development of organelles during seed germination is suggested in *Arabidopsis* transgenic lines with *MGD1* expression suppressed by *amiR*-*MGD1* in a dexamethasone (DEX)-dependent manner (Fujii et al. 2014). The induction of *amiR*-*MGD1* by DEX treatment reduced *MGD1* transcript levels in cotyledons by 75 % as compared with the untreated control level, thereby greatly decreasing MGDG biosynthesis and galactolipid content. As observed in the *mgd1*-*2* mutant (Kobayashi et al. 2013), galactolipid deficiency in DEX-treated *amiR*-*MGD1* seedlings impaired thylakoid membrane biogenesis and decreased the expression of photosynthesis-associated genes encoded in plastids and the nucleus. Along with photosynthesis-associated genes, genes involved in photorespiratory metabolism in peroxisomes and mitochondria were downregulated by *MGD1* suppression (Fujii et al. 2014). By contrast, the expression of genes associated with the glyoxylate cycle, markedly decreased during early cotyledon development in the wild type (Graham 2008), remained at high levels in DEX-treated *amiR*-*MGD1* seedlings independently of triacylglycerol levels in cotyledons (Fujii et al. 2014). The high expression of glyoxylate cycle-associated genes with low expression of photorespiratory genes in response to the *MGD1* suppression suggest that galactolipid biosynthesis and subsequent thylakoid biogenesis somehow affect the differentiation of peroxisomes and mitochondria along with chloroplast differentiation at transcription levels.

In addition, loss of thylakoid lipid biosynthesis strongly affects mesophyll cell organization and leaf development. The *mgd1*-*2* mutant showed stunted embryogenesis at the globular stage and failed to develop cotyledons after germination (Kobayashi et al. 2007; Kobayashi and Ohta 2008). Although *mgd1*-*2* could develop true leaves slowly under nutrient-sufficient conditions with sucrose supplementation, the development of mesophyll cells was severely perturbed, with distorted epidermal cell layers and large intercellular spaces within leaves (Fig. 3) (Kobayashi et al. 2007). However, disordered cell development in the mutant leaves was recovered to some extent, along with internal membrane biogenesis in plastids, under P-deficient conditions (Fig. 3). Moreover, Fujii et al. (2014) reported in *amiR*-*MGD1* lines that blocking MGDG biosynthesis by *MGD1* suppression impaired post-germinative growth of both epidermal and mesophyll cells in cotyledons, as illustrated in their irregular shapes and disordered alignment with indented cell outlines. These data suggest that galactolipid biosynthesis and consequent thylakoid membrane development are essential for ordered cell development during embryogenesis, true leaf biogenesis, and even post-germinative development of cotyledons.

Anionic thylakoid lipids also play an important role in leaf cell development. In the *pgp1*-*2* mutant grown under nutrient-sufficient conditions, leaf mesophyll cells developed only around vascular structures, so large intercellular spaces were observed between mesophyll cells as in the *mgd1*-*2* mutant (Fig. 3) (Hagio et al. 2002; Kobayashi et al. 2015). Epidermal cell layers were also distorted and abnormally crooked in the *pgp1*-*2* leaves. However, as in *mgd1*-*2*, in *pgp1*-*2* leaves, intercellular spaces were decreased and the distortion of epidermal cell layers was moderated with P deficiency (Fig. 3) (Kobayashi et al. 2015). Because photosynthetic activity was entirely abolished both in *pgp1*-*2* and *mgd1*-*2* under P-deficient conditions, glycolipid accumulation and thylakoid membrane biogenesis would lead to leaf cell development independent of photosynthesis. Furthermore, even a 30 % reduction in total PG content in the leaky *pgp1*-*1* mutant caused decreased number of mesophyll cells in leaves, although the mutation did not markedly affect thylakoid membrane development inside leaf chloroplasts (Xu et al. 2002; Yu and Benning 2003). The additional mutation of *sqd2* to *pgp1*-*1* further decreased the number of mesophyll cells and increased the intercellular spaces in leaves (Yu and Benning 2003). These data indicate a special requirement for anionic lipids for differentiation, development and alignment of mesophyll cells besides their roles in photosynthesis. A set of evidence suggests that plastid signaling plays a role in coordinating the development of leaf cells with chloroplast functionalities (Andriankaja et al. 2012; Ruckle and Larkin 2009), so lipid biosynthesis in chloroplasts may be involved in coordinated development of chloroplasts and leaf cells via plastid signaling pathways.

## Coordinated regulation of galactolipid biosynthesis with formation of photosynthetic machinery

Development of the thylakoid membrane during chloroplast biogenesis is one of the most remarkable changes within leaf cells and requires a substantial increase in content of galactolipids along with other photosynthetic components. Several studies suggest that the biosynthesis of galactolipids is coordinated with chlorophyll and photosynthetic protein synthesis and the formation of photosynthetic machinery.

In cucumber cotyledons, MGDG synthesis activity and galactolipid content were notably increased after illumination of etiolated seedlings (Ohta et al. 1995b; Yamaryo et al. 2003). At the same time, the expression of the cucumber *MGD1* (*csMGD1*) in cotyledons was rapidly and transiently induced in response to light (Yamaryo et al. 2003). However, the acute response of *csMGD1* expression to light was attenuated in cotyledons detached at the hook before illumination, which suggests that light is not sufficient for full upregulation of *csMGD1* and requires other factors presumably transported from the hypocotyl or roots. Moreover, exogenous cytokinin treatment to detached cotyledons induced *csMGD1* expression in the dark (Yamaryo et al. 2003). Therefore, cytokinin, which may be transported from the hypocotyl or roots to cotyledons, may play an important role in the upregulation of *csMGD1* in response to light. Meanwhile, in detached cotyledons, galactolipid synthesis was still increased by illumination despite the impaired *csMGD1* expression, so light may activate galactolipid biosynthesis in a post-transcriptional manner (Yamaryo et al. 2003). Moreover, exogenous cytokinin treatment to detached cotyledons moderately increased galactolipid content in the dark (Yamaryo et al. 2003). Taken together, the great accumulation of galactolipids for chloroplast development during cotyledon greening may require transcriptional activation of *csMGD1* by cytokinin signaling and post-transcriptional activation of MGDG synthesis by light in an additive manner.

Further analysis of post-translational regulation of MGD1 in vitro revealed that the enzymatic activity of MGDG synthase was increased by chloroplast thioredoxins in a redox-dependent manner (Shimojima et al. 2013; Yamaryo et al. 2006). Thioredoxins contain a redox-reactive cysteine pair within the active site and facilitate the reduction of target proteins by cysteine thiol–disulfide exchange. Various biological processes during chloroplast development such as transcription, translation, carbon metabolism including the Calvin cycle, synthesis of chlorophylls and fatty acids, and assembly of photosynthetic protein-cofactor complexes are regulated by the thioredoxin redox system in a light-dependent manner (Geigenberger et al. 2005; Serrato et al. 2013). Yamaryo et al. (2006) reported that csMGD1 activity in vitro was reversibly regulated by reduction and oxidation via an intramolecular disulfide bond(s). Two chloroplast thioredoxins, Trx-f and Trx-m, efficiently reduced disulfide bonds of csMGD1 and increased its activity. In addition to MGDG biosynthesis, fatty acid synthesis is also activated in response to light via thioredoxin-mediated redox regulation of acetyl-CoA carboxylase, which catalyzes the first committed step of *de novo* fatty acid synthesis (Sasaki et al. 1997). Therefore, galactolipid synthesis and *de novo* fatty acid synthesis may be linked with photosynthetic electron transport via the thioredoxin-mediated redox regulation at post-translational levels.

In addition to redox regulation, the anionic phospholipids PA and PG modify enzymatic activity of MGDG synthases (Covés et al. 1988; Dubots et al. 2010; Ohta et al. 1995a; Shimojima et al. 2013). Although purified *Arabidopsis* MGD1 had no enzymatic activity in the absence of membrane lipids in the reaction mixture, the addition of PA at a very low concentration sufficiently activated MGD1 enzyme (Dubots et al. 2010). The addition of PG also increased MGD1 activity but with a higher concentration than PA. Dubots et al. (2010) demonstrated that PA and PG can activate MGD1 in a synergistic manner via different pathways. The authors further revealed that MGDG synthase activity in leaf homogenates was decreased in the knockout mutant of a phospholipase D gene (*PLDz2*) probably because of reduced PA levels, which suggests the importance of PA metabolism for MGDG synthase activity in vivo. As discussed in Botella et al. (2016), intracellular phospholipid metabolism may be linked with the regulation of MGDG synthase activity via modulating local PA levels. In addition, Sarkis et al. (2014) revealed that the *Arabidopsis* MGD1 protein is attracted to artificial lipid monolayers enriched with MGDG but not those enriched with DGDG. Moreover, the presence of PG in the MGDG:DGDG mixture increases the binding of MGD1 to the lipid monolayer presumably via electrostatic interactions (Sarkis et al. 2014). A crystallographic analysis and mutational studies of the catalytic domain of MGD1 support the hypothesis that PG contributes to anchoring MGD1 at the membrane surface and helping to bring diacylglycerol close to UDP-galactose in the active site (Rocha et al. 2016). These data suggest that local lipid composition in the plastid envelope may affect MGDG synthase activity by modifying the localization and affinity of the MGD1 protein to the envelope.

An importance of post-translational regulation of MGDG synthase for chloroplast development is suggested by a heterogenic complementation of the *Arabidopsis* *mgd1*-*2* mutant with the MGDG synthase gene (*mgdA*) from the green sulfur bacterium *Chlorobaculum tepidum*. Masuda et al. (2011) reported that complemented *mgd1*-*2* plants expressing *C. tepidum* *mgdA* showed highly disorganized thylakoid formation in some chloroplasts, although the transgenic plants accumulated galactolipids to near wild-type levels. Considering that MgdA from *C. tepidum* has no conserved cysteine residues for redox regulation, with its activity unaffected by PA addition (Masuda et al. 2011), heterogenic *mgdA* expression may cause dysregulation of MGDG synthesis in *mgd1*-*2* chloroplasts and lead to the aberrant thylakoid formation.

In addition to post-translational regulation, transcriptional regulation would play a role in coordinating galactolipid biosynthesis with chloroplast development. Most photosynthesis-associated genes are light-inducible, and the expression of *MGD1* and *DGD1* in *Arabidopsis* was increased in response to light (Awai et al. 2001; Kobayashi et al. 2014). A basic Leu zipper transcription factor, HY5, which plays a pivotal role in photomorphogenesis and photosynthetic gene expression downstream of photoreceptors (Bae and Choi 2008), is essential for the upregulation of *MGD1* and *DGD1* in response to light (Kobayashi et al. 2014). *DGD1* but not *MGD1* has been identified as a putative direct target of HY5 (Lee et al. 2007), so regulatory pathways in response to light may differ between *MGD1* and *DGD1*. In addition, the light-inducible expression of *MGD1* and *DGD1* was impaired in a cytokinin receptor mutant, which suggests that cytokinin signaling is also required for the light response of these galactolipid genes (Kobayashi et al. 2014). Moreover, the expression of *MGD1* and *DGD1* in etiolated *Arabidopsis* seedlings was slightly but significantly increased along with that of other photosynthesis-associated genes by cytokinin treatment, which is consistent with the report in cucumber (Yamaryo et al. 2003). GOLDEN2-LIKE (GLK) transcription factors, which directly upregulate genes involved in chlorophyll biosynthesis and light harvesting (Waters et al. 2009), also affect the expression of galactolipid biosynthesis genes, particularly *DGD1* (Kobayashi et al. 2014). These regulatory factors associated with chloroplast biogenesis may play an important role in the coordinated formation of the thylakoid lipid bilayer with photosynthetic machineries at the transcriptional level.

Galactolipid biosynthesis activities strongly affect the expression of genes involved in photosynthesis and chlorophyll biosynthesis; chlorophyll biosynthesis and chloroplast functionality also affect the expression of galactolipid synthesis genes. In fact, the expression of *MGD1* and *DGD1* was decreased in mutants deficient in chlorophyll biosynthesis (Kobayashi et al. 2014). Moreover, impaired chloroplast development by norflurazon treatment downregulated *MGD1* and *DGD1* along with other photosynthesis-associated genes (Kobayashi et al. 2014). These data indicate that the expression of *MGD1* and *DGD1* is coordinated with the formation of photosynthetic machineries and chloroplast development. Because the mutant for GENOMES UNCOUPLED 1 (GUN1), a central regulator of plastid signaling (Koussevitzky et al. 2007), showed only weak downregulation of *MGD1* and *DGD1* on norflurazon treatment, the expression of these galactolipid genes is under plastid signal regulation, with GUN1 playing a pivotal role (Kobayashi et al. 2014). Consistent with these data, the expression of *MGD1* and *DGD1* was downregulated in the *pgp1*-*2* mutant, which failed to develop functional chloroplasts (Kobayashi et al. 2015). However, the expression of *MGD1* and *DGD1* in *pgp1*-*2* was increased with P deficiency (Kobayashi et al. 2015). Because P deficiency itself does not induce *MGD1* expression in wild-type *Arabidopsis* (Awai et al. 2001; Kobayashi et al. 2004), chloroplast biogenesis in the P-deficient *pgp1*-*2* may positively affect *MGD1* expression (Kobayashi et al. 2015). The *MGD1* and *DGD1* expression and subsequent galactolipid biosynthesis induced thylakoid biogenesis and chloroplast development, whereas thylakoid biogenesis and chloroplast development upregulated these galactolipid genes. Therefore, the expression of galactolipid genes and thylakoid formation may be affected reciprocally during chloroplast biogenesis.

## Perspectives

In addition to early biochemical studies, recent advances in molecular biological studies with genetic and reverse genetic approaches in plants, algae and cyanobacteria have allowed for elucidating the biosynthetic pathways and physiological roles of thylakoid lipids. Moreover, recent studies have suggested an involvement of thylakoid lipid biosynthesis in the coordinated formation of photosynthetic machineries during chloroplast biogenesis, which would guarantee the efficient formation of photosynthetic systems while preventing the unbalanced accumulation of thylakoid constituents and intermediates that are potential sources of photooxidative stresses.

Meanwhile, a number of questions about thylakoid lipid metabolism remain, particularly in terms of trafficking and regulation. The lipid trafficking mechanism for thylakoid membrane biogenesis (e.g., via envelope invagination, vesicle transport) needs to be elucidated to understand how entire sets of photosynthetic complexes are assembled with thylakoid lipids during chloroplast biogenesis. In addition, information on transcriptional and post-translational regulators involved in thylakoid lipid biosynthesis is severely deficient as compared with the increasing knowledge about regulators for chlorophyll and photosynthetic protein synthesis. The molecular mechanism linking the functional state of lipid biosynthesis with photosynthesis-associated gene expression in plastids and nucleus awaits future studies.
