# Correction to: Role of membrane glycerolipids in photosynthesis, thylakoid biogenesis and chloroplast development

**DOI:** 10.1007/s10265-018-1011-3

**Published:** 2018-02-21

**Authors:** Koichi Kobayashi

**Affiliations:** Department of Life Sciences, Graduate School of Arts and Sciences, The University of Tokyo, Komaba 3-8-1, Meguro-ku, Tokyo, 153-8902 Japan

## Correction to: J Plant Res (2016) 129:565–580 10.1007/s10265-016-0827-y

The article “Role of membrane glycerolipids in photosynthesis, thylakoid biogenesis and chloroplast development”, written by “Koichi Kobayashi”, was originally published Online First without open access. After publication in volume 129, issue 4, page 565–580 the Botanical Society of Japan decided to opt for Open Choice and to make the article an open access publication. Therefore, the copyright of the article has been changed to © The Author(s) 2018 and the article is forthwith distributed under the terms of the Creative Commons Attribution 4.0 International License (http://creativecommons.org/licenses/by/4.0/), which permits use, duplication, adaptation, distribution and reproduction in any medium or format, as long as you give appropriate credit to the original author(s) and the source, provide a link to the Creative Commons license, and indicate if changes were made.

